# Geriatric nutritional risk index and quality of life among elderly hemodialysis patients: a cross-sectional study

**DOI:** 10.1097/MS9.0000000000002378

**Published:** 2024-07-17

**Authors:** Ali Nouri, Roya Mansour-ghanaei, Mohammad Esmaeilpour-Bandboni, Bahare Gholami Chaboki

**Affiliations:** a Zeynab (P.B.U.H) School of Nursing and Midwifery, Guilan University of Medical Sciences; b Gastrointestinal and Liver Diseases Research Center, Guilan University of Medical Sciences, Rasht, Iran

**Keywords:** aged, geriatric nutritional risk index, quality of life, renal dialysis

## Abstract

**Background:**

Nutritional problems are considered one of the main complications of hemodialysis, and the geriatric nutritional risk index (GNRI) is a new instrument for assessing geriatric nutritional status. The present study sought to evaluate the relationship between the GNRI and quality of life (QOL) among elderly hemodialysis patients.

**Methods:**

In the present analytical cross-sectional study, 110 hemodialysis individuals were selected by applying a simple random sampling method, among whom 57 and 53 were males and females, respectively (mean: 70.3±6.93 years). Demographic characteristics, GNRI, and QOL status were determined. The data were analyzed using SPSS 20 software and descriptive statistics, Pearson’s correlation, ANOVA, independent sample *t*-tests, and multiple linear regression analysis.

**Results:**

The mean GNRI and mean total QOL were respectively obtained 93.903±11.067 and 20.95 ± 4.89. Among females, a significant direct relationship was observed between GNRI and total QOL (*P* = 0.010, r = 0.352), autonomy (*P* = 0.004, r = 0.389), and pleasure (*P* = 0.015, r = 0.333), while GNRI was not directly and significantly related to QOL in males (*P* = 0.161, r = 0.188).

**Conclusion:**

Due to the presence of a significant association between Geriatric GNRI and QOL among elderly hemodialysis patients, particularly among females, highlighting the importance of addressing nutritional status in optimizing well-being.

## Introduction

Highlights

- Hemodialysis patients experience varying degrees of reduced quality of life.
- Quality of life (QOL) is considered as one of the important challenges in the healthcare field and the main factor in the survival at chronic diseases.
- Geriatric nutritional risk index (GNRI) can be applied as an instrument determinant the QOL of hemodialysis patients.

Chronic kidney disease (CKD), causes kidney failure and leads to hemodialysis and kidney transplantation, is considered one of the main medical challenges and is an important public health problem, that is spreading worldwide^1,2^. Around 5–7% of the world’ population suffers from the types of CKDs^3^, the prevalence of which increases with age to changes in the structure and function of kidneys^4^. In addition, about 45% of the elderly are affected by different degrees of dysfunction, which eventually leads to end-stage kidney disease (ESKD)^5^. The disease causes various problems such as weakness, bone pain, weight loss, itching, and nausea, as well as a loss of appetite, which influence patients’ quality of life (QOL)^6^. In Iran, there are ~39 000 ESKD hemodialysis patients,^7^ with ~15% being added each year^8^. Hemodialysis individuals experience different decreases in QOL^9^, one reason for which includes the reduced musculoskeletal mass induced by hemodialysis, which exposes them to various fractures and declines their QOL^10^. Further, the adherence of the patients to hemodialysis treatment plans, usually three times a week (about 15 h), results in disrupting their normal lives and diminishing their QOL^11^. QOL is among the crucial challenges in the healthcare field and one of the main factors in survival, as well as the therapeutic outcome of chronic diseases^6^. In fact, it is a broad concept, that covers the various aspects of life, such as religion, love, belonging, financial and occupational status, and mental, social, and physical health^12^. Furthermore, QOL is a predictor of mortality in ESKD patients and the most important criterion for determining their health outcomes. The QOL assessment can be effective in diagnosing, predicting, evaluating, and implementing efficient care and treatment methods to help individuals with chronic diseases and even healthy ones^13^. A significant reduction in nutritional variables like weight, muscle, and fat mass can be addressed as another complication of hemodialysis^14^, leading to protein-energy loss^15^. Protein-energy loss is known as one of the factors affecting mortality growth among kidney patients. Examining and managing the nutritional status of such individuals play a crucial role in preventing the complications of kidney diseases and hemodialysis^16^. The geriatric nutritional risk index (GNRI) has been suggested as an instrument for measuring the risk of nutritional complications, and a predictor for mortality among the elderly^15^. Lis *et al*.^17^ proposed individuals’ nutritional status as an efficient predictor of QOL in cancer patients. Kang *et al*.^18^ reported that this index can be effectively utilized to forecast mortality in individuals receiving peritoneal dialysis. According to Matsumura *et al*.^19^, GNRI is positively related to sarcopenia among chronic obstructive pulmonary disease patients. Given the significant effect of this treatment method on the QOL of hemodialysis patients as well as the decline in their life expectancy, it is considered important to assess the QOL-related factors and try to improve QOL. As a result, the current study sought to specify the relationship between the GNRI and QOL in elderly hemodialysis patients.

## Materials and methods

### Study design and patient population

This analytical cross-sectional study was conducted among 110 individuals of 60 years of age or older admitted to the hemodialysis centers of Guilan Province, who were selected based on a simple random sampling method. Before providing consent, the Participants were informed about the objectives, implementation method, benefits, and potential risks, as well as familiarized with the instruments used. Additionally, the medical history of all patients was obtained by using medical records, and interviewing the individuals and their families. The inclusion criteria included age older than 60 years and above, appropriate mental and cognitive ability, informed consent to participate in this study, as well as at least a 6-month history of hemodialysis. Further, the lack of malignancy, autoimmune diseases, and muscle movement disorders based on the medical record, no history of amputation, and non-consumption of corticosteroids during the study period were the other inclusion criteria. However, those withdrawing from the study were excluded. The sample size was estimated at 91 based on the study of Beberashvil (2016) (*P* = 0.05, r = 0.29, power = 0.8), which was enhanced to 110 by considering the 20% probability of sample loss.

### Data collection

After selecting the eligible elderly patients, they filled out informed consent forms and completed a demographic information questionnaire, as well as the CASP-19 quality of life scale for older people. The first consists of some questions on gender, age, education level, occupation, dialysis duration, marital and financial status, as well as the place and status of residence and the history of underlying diseases. Hyde and colleagues based on the needs satisfaction model designed the CASP-19 quality of life scale for older people, the second questionnaire. Heravi-Karimooi and colleagues standardized this questionnaire in Iran and reported the Cronbach’s alpha and correlation index of 0.7–0.88 and 0.97<*P*<0.001 for its Persian version. The CASP-19 comprises four subscales of control, autonomy, self-realization, and pleasure, the item numbers of which is 4, 5, 5, and 5, respectively (19 items). Each item is scored based on a four-point Likert scale (0=never to 3=often). Thus, the maximum and minimum scores of the questionnaire are 57 (full satisfaction with all subscales) and 0 (complete lack of QOL), respectively^20^.

The GNRI was computed through measuring the current and ideal weight, height, and albumin level of the individuals. The Participants were divided into four groups based on their GNRI: severe, moderate, mild, and no nutritional risk (GNRI 82, 82>92, 92–98, and >98, respectively)^21^.

### Ethical approval

Ethical committee of Guilan University of Medical Sciences approved this study (ethics code: IR.GUMS.REC.1399.113). Informed consent was obtained from all the participants. This study was conducted in accordance with the Helsinki declaration. This study adheres to the STROCSS (Strengthening the Reporting of Cohort Studies in Surgery) criteria^22^, for comprehensive and transparent reporting of surgical cohort studies.

### Statistical analysis

The data were analyzed using descriptive (frequency distribution tables, mean, and standard deviation) and inferential statistics (independent sample *t*-tests, ANOVA, Pearson’s correlation, and multiple linear regression) in SPSS 20 software. Furthermore, the normality of the study variables was examined through the Kolmogorov–Smirnov test. In all tests, P 0.05 was considered the significance level.

## Results

In the present study, 110 elderly hemodialysis patients with a mean age of 70.3 ± 6.93 years were assessed, and the relevant demographic characteristics and their relationship with QOL are summarized in Table 1. Additionally, the mean (SD), maximum, and minimum of GNRI were respectively obtained 93.9 (11.06), 72.36, and 122.59 years, which 37.3, 47.3, and 15.5% of the individuals were placed in no, mild-moderate, and severe nutritional risk, respectively. The mean total QOL score was equal to 20.95 ± 4.89 and most of the Participants acquired scores less than the moderate level, which indicates the low QOL of elderly hemodialysis patients.

**Table 1:** Demographic characteristics and their relationship with QOL among hemodialysis patients (*n*=110)

Variable	Variable level	*N* (%)	QOL	*P*
Sex	Male	57 (51.8)	20.05±4.98	0.04
	Female	53 (48.2)	21.92±4.65	
Age	60–74 years	79 (71.8)	21.72±4.61	0.00
	75–85 years	31 (28.2)	19±5.11	
Marital status	Married	67 (60.9)	21.49±4.68	0.38
	Single	43 (39.1)	20.11±5.15	
Education level	Illiterate	79 (71.8)	20.43±4.49	0.07
	Literate	31 (28.2)	22.29±5.65	
Financial adequacy	Adequate	52 (43.7)	20.76±4.10	0.71
	Inadequacy	58 (52.7)	21.12±5.54	
No. children	≤3	62 (56.4)	21.17±4.35	0.58
	≥4	48 (43.6)	20.66±5.55	
Life status with	Single	14 (12.7)	19.78±5.01	0.49
	Wife	66 (60)	21.24±4.74	
	Children	30 (27.3)	20.86±5.25	

QOL, quality of life.

Table 2 represents the results of the mean (SD) of QOL, as well as its subscales and relationship with GNRI.

**Table 2:** Relationship between GNRI with QOL and its subscales among the elderly patients undergoing hemodialysis (*n*=110)

Criterion	Mean (SD)	Correlation (r) with GNRI	Level of significance
Total QOL	20.95±4.89	0.07	0.46
Control subscale	5.98±2.47	0.03	0.71
Autonomy subscale	7.06±1.69	0.10	0.26
Self-realization subscale	2.70±2.05	0.05	0.54
Pleasure subscale	5.20±2.88	0.09	0.32

GNRI, geriatric nutritional risk index; QOL, quality of life.

The relationship between GNRI and QOL and its subscales by gender is presented in Table 3, which demonstrates no significant relationship. However, the GNRI of females was directly and significantly related to their QOL, as well as the subscales of autonomy and pleasure (*P* = 0.010, r = 0.352).

**Table 3:** Relationship between GNRI with QOL and its subscales among elderly hemodialysis patients by sex (*n*=110)

Sex	Criterion	Mean (SD)	Correlation (r) with GNRI*	Level of significance
Male				
	Total QOL*	20.05±4.98	0.18	0.1
	Control subscale	6.70±2.36	0.01	0.94
	Autonomy subscale	7.10±1.80	−0.15	0.24
	Self-realization subscale	2.40±2.19	−0.13	0.33
	Pleasure subscale	3.84±2.56	−0.11	0.40
Female	Total QOL	21.92±4.65	0.35	0.01*
	Control subscale	5.20±2.37	0.02	0.85
	Autonomy subscale	7.01±1.57	0.38	0.004*
	Self-realization subscale	3.01±1.86	0.01	0.95
	Pleasure subscale	6.67±2.47	0.33	0.01*

GNRI, geriatric nutritional risk index; QOL, quality of life.

## Discussion

The present study determined the relationship between the GNRI and the QOL of hemodialysis patients. The results revealed a significant relationship between gender and QOL, such that the mean QOL was higher among elderly female hemodialysis patients compared to males. This issue can be attributed to the higher access of females to social support instruments in comparison with males, which has an effect on enhancing their resilience to problems^23^. Another reason includes the difference in lifestyles between males and females. In fact, males possess a more dangerous and unhealthy lifestyle, and consume more cigarettes and alcohol compared to females, leading to a decrease in their QOL. In addition, the prevalence of unbearable diseases such as cancer and stroke is higher among males, which affects their QOL significantly^24,25^.

Based on the results of the present study, the QOL of 60–74 year patients was twofold higher than that of the older Participants, which is in line with the results of Taheri *et al*.^12^ and Daniela *et al*.^26^. Age is considered one of the important factors in QOL, and an increase in age results in growing rates of chronic diseases such as diabetes, kidney failure, cancer, and stroke, as well as declining QOL at age^27^.

The different lifestyles, diets specific to each country, and climate conditions in each region are among the factors determining the nutritional quality of individuals, causing the inconsistency between the results of the present study and the ones mentioned above.

The findings of this study revealed a low level of QOL among elderly hemodialysis patients. Given that the mean QOL score was 20.95 ± 4.89 and the maximum achievable score was 57 in the questionnaire, most of the Participants had a QOL below the moderate level.

The result is in agreement with those of some studies that reflected low QOL among hemodialysis patients^28–30^. In fact, the normal life of hemodialysis patients faces a crisis since the function of their kidney, as one of the vital organs of the body, is lost. Thus, they get hemodialysis, which puts them in an unpleasant situation and influences their QOL negatively and significantly due to the aggressive, long, and time-consuming nature of the procedure.

GNRI was not significantly correlated with QOL and its subscales in males. However, a direct and significant relationship was attained between GNRI and total (*P* = 0.01, r = 0.352), autonomy (*P* = 0.004, r = 0.389), and pleasure (*P* = 0.015, r = 0.333) in females. The present study is considered as the second research project measuring the relationship between the two variables. The results of this study are not in line with those of Beberashvili and colleagues, which demonstrated a direct relationship between GNRI and total QOL among both elderly hemodialysis males and females in Israel (*P* = 0.15). Among the subscales of QOL, this index is directly and significantly related to physical health (*P* = 0.14)^31^.

The geographic and demographic difference between the Participants of the two studies is one of the main reasons for the contradictory results. Indeed, various cultures, lifestyles, and beliefs are observed in the two studies, which have a significant effect on how people deal with problems and diseases, as well as their QOL. Furthermore, females usually follow a more useful and healthier diet than males. Their food basket mainly contains fruit and vegetables, while males often tend to eat fats and sweets^32^. The consumption of healthier foods by females can be among the possible reasons for the direct relationship between GNRI and better QOL compared to males.

The limitations of this study included the difficulty of communicating with the Participants and gathering the data due to the COVID-19 pandemic. In this regard, attempts were made to prevent the disruption of the study by observing safety tips to the maximum.

The results of this study and similar studies, paves the way for the development of novel methodologies for the prompt and continuous assessment of QOL in elderly populations. This information holds significant promise for informing the development of targeted programs and policies aimed at improving the well-being of elderly people. Moreover, by establishing a more comprehensive approach to nutritional assessments, this study lays the groundwork for a paradigm shift in the care of elderly hemodialysis patients.

## Conclusion

This research delves into the intricate connection between geriatric nutritional status, measured by GNRI, and QOL in elderly hemodialysis patients, stressing the importance of addressing nutritional needs in this group. Particularly among females, a significant link was found between GNRI and various QOL domains, highlighting the potential of nutritional interventions to enhance well-being and life satisfaction. The absence of a similar correlation in males suggests a complex interplay of factors affecting QOL, necessitating further exploration. Tailored nutritional management strategies are crucial for optimizing QOL and overall health outcomes in elderly hemodialysis patients. Future longitudinal studies are needed to understand the causal links and mechanisms underlying these associations, guiding targeted interventions to improve the well-being of this vulnerable population.

## Ethical approval

This study was approved by ethical committee of Guilan University of Medical Sciences (ethics code: IR.GUMS.REC.1399.113). Informed consent was obtained from all the participants. This study was conducted in accordance with the Helsinki declaration.

## Consent

Not applicable.

## Source of funding

This research did not receive any specific grant from funding agencies in the public, commercial, or not-for-profit sectors.

## Author contribution

All authors have made substantial contributions to the following: R.M.G., A.N. and M.E. designed the research; A.N. carried out the data collection; R.M.G., M.E., and B.G. completed the data and statistical analyses and drafted the manuscript; all authors edited, read, and approved the final manuscript.

## Conflicts of interest disclosure

The authors declare that they have no conflicts of interest.

## Research registration unique identifying number (UIN)

The ethical committee of Guilan University of Medical Sciences (IR.GUMS.REC. 1399.113) approved the study.

https://ethics.research.ac.ir/ProposalCertificateEn.php?id=137448&Print=true&NoPrintHeader=true&NoPrintFooter=true&NoPrintPageBorder=true&LetterPrint=true.

## Guarantor

Roya Mansour-ghanaei.

## Data availability statement

The datasets generated during and/or analyzed during the current study are available upon reasonable request.

## Provenance and peer review

Not commissioned, externally peer-reviewed.

## Availability of data and materials

The dataset is available from the corresponding author upon request.
